# Factors associated with early-onset androgenetic alopecia: A scoping review

**DOI:** 10.1371/journal.pone.0299212

**Published:** 2024-03-07

**Authors:** Li-Ping Liu, Mary Adumo Wariboko, Xiao Hu, Zi-Han Wang, Qian Wu, Yu-Mei Li

**Affiliations:** 1 Department of Dermatology, Affiliated Hospital of Jiangsu University, Zhenjiang, Jiangsu, China; 2 Institute of Regenerative Medicine, Jiangsu University, Zhenjiang, Jiangsu, China; 3 Department of Urology, The First People’s Hospital of Zhenjiang, Zhenjiang, Jiangsu, China; University of Warwick, UNITED KINGDOM

## Abstract

**Background:**

Early-onset androgenetic alopecia (AGA) has been associated with various chronic conditions, including metabolic syndrome (MetS). Gaining a deep understanding of early-onset AGA may enable earlier intervention in individuals at high risks. This scoping review aims to explore the risk factors and etiology, associated conditions, and adverse effects on wellbeing in early-onset AGA.

**Methods:**

Electronic literature searches were conducted in MEDLINE, EMBASE and CENTRIAL. Eligible studies included case-control, cohort, cross-sectional, and meta-analysis studies. Selected studies needed to clearly define early-onset AGA cases or include only cases starting before the age of 40 and compare them with appropriate controls. The exclusion criteria comprised editorials, commentaries, case series, and non-systematic reviews, among others. Data extraction involved collecting study characteristics, methodologies, main outcomes, and findings. Descriptive tables were used to summarize key information and relevant variables when necessary.

**Results:**

Among the 65 eligible articles, 67.69% were case-control studies and 78.46% evaluated only male patients. “Early-onset” was defined as cases developing before the age of 30 years in 43.08% of the studies. The Hamilton–Norwood scale was the most frequently used method for evaluating the severity of alopecia in men (69.23%). Reported risk factors for early-onset AGA included a family history of AGA, cigarette smoking, unhealthy dietary habits, and a high body mass index. Early-onset AGA may also be associated with hormonal profiles, 5α-reductase enzyme activity, androgen receptor genes, and some susceptibility loci. Comorbidities investigated included MetS, cardiovascular disease, insulin resistance, dyslipidemia, and Parkinson’s disease. Men with early-onset AGA may have reduced treatment efficacy with drug like rosuvastatin, metformin or lisinopril for dyslipidemia, prediabetes, or hypertension. Additionally, young men with AGA tended to suffer from psychological issues such as anxiety and low self-esteem compared to those without hair loss.

**Conclusion:**

Early-onset AGA is a complex condition with various risk factors and etiology, associated comorbidities, and potential implications for treatment response and psychological health.

## Introduction

Androgenetic alopecia (AGA) is the most prevalent cause of non-scarring alopecia in adults, typically affecting the front and vertex regions in men and causing diffuse hair thinning over the central scalp in women [1]. The severity of AGA is commonly evaluated using the Hamilton–Norwood scale (HNS) in men [2] and the Ludwig classification in women [3]. Early-onset AGA is generally defined as AGA developing before the age of 30 [4, 5], 35 [6, 7] or 40 [8, 9] years; however, there is currently no unified definition. Although the frequency of AGA increases with age, the prevalence of early-onset cases is substantial, ranging from 19.2%-57.6% in different populations [10–12]. In addition to harmfully affecting the patient’s appearance and quality of life [13, 14], AGA is closely associated with the risk of developing metabolic syndrome (MetS) [15, 16], which is a cluster of metabolic abnormalities including obesity, hyperlipidemia, insulin resistance (IR), and hypertension. A systematic review including 19 articles and 2,531 participants revealed a pooled odds ratio (OR) of 3.46 for the prevalence rate of MetS between individuals with AGA and controls [15]. Moreover, early-onset AGA has been associated with early markers of IR [6], carotid artery atherosclerosis [17], ischemic heart disease [18], and benign prostatic hyperplasia [19].

Considering the detrimental consequences and burden associated with these chronic disorders, more comprehensive characterization and in-depth understanding of early-onset AGA could aid in identifying individuals at high risk. This, in turn, would facilitate improved and earlier interventions, ultimately preventing long-term consequences during the early stages of life. This review aims to scope, collate and catalogue the existing literature on early-onset AGA. Specially, the focus will be on identifying risk factors and etiology, associated comorbidities and potential adverse effects on overall wellbeing.

## Methods

We used the Preferred Reporting Items for Systematic reviews and Meta-Analyses extension for Scoping Reviews (PRISMA-ScR) [20] for reporting this scoping review (S1 Table) and reported the flow of articles included in accordance with the Checklist. The study’s protocol was not previously published.

### Research questions

The scoping review was guided by the following specific research questions. First, what are the risk factors associated with early-onset AGA? Second, what comorbidities are individuals with early-onset AGA at a higher risk of developing? Third, are there any additional adverse effects on patient well-being associated with early-onset AGA?

### Search strategy

Electronic literature searches were performed in MEDLINE, EMBASE and CENTRIAL on July 28, 2023. The searches were conducted for studies published in the English language, without imposing any specific restrictions on the publication period. The search terms used in the database are shown in S2 Table. EndNote citation management software was utilized for managing the literature.

### Study selection

**Fig 1:**
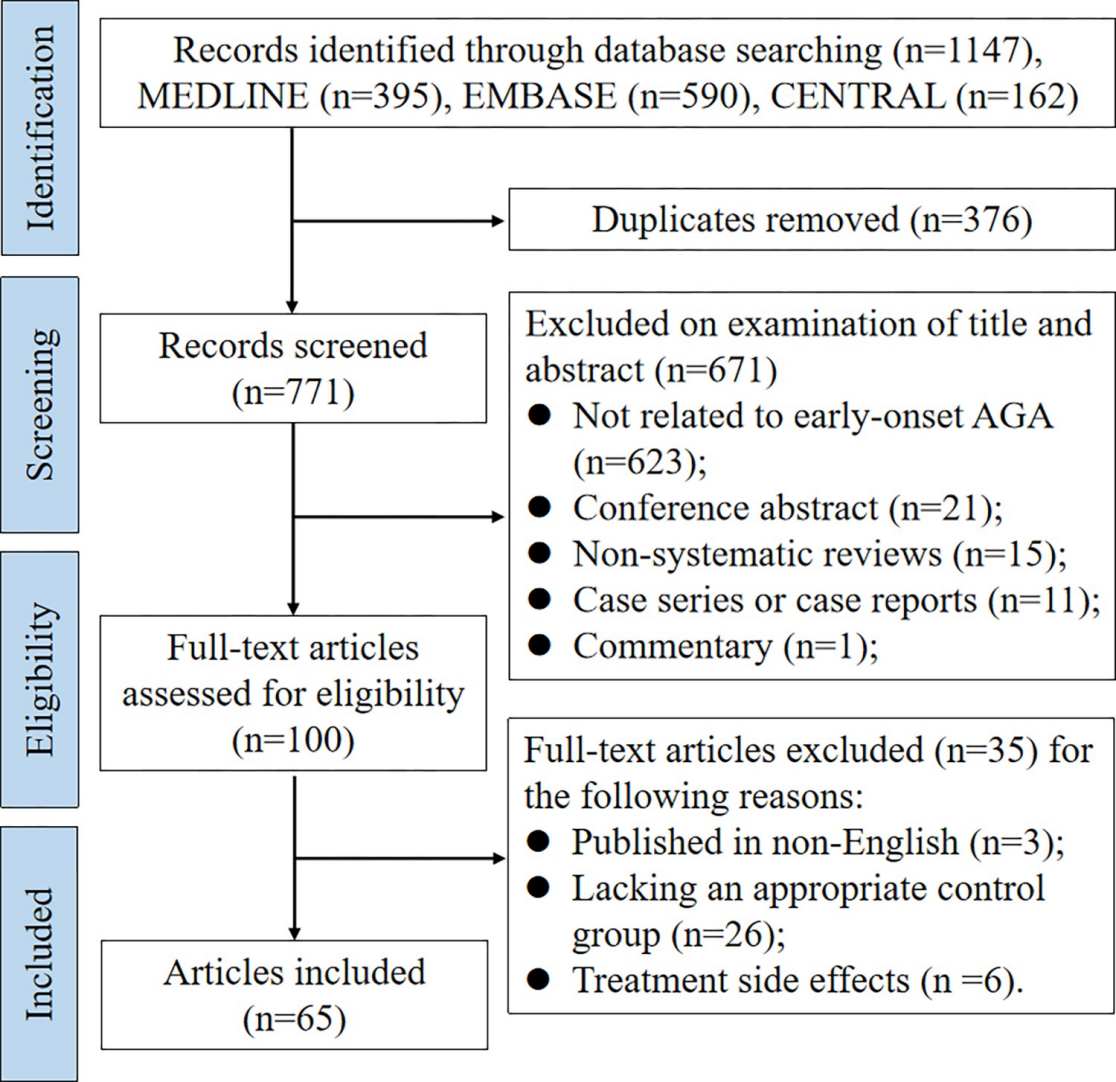
Search strategy and study selection.

The search strategy is shown in Fig 1. The inclusion criteria for articles were as follows: 1) Research articles based on case control, cohort, cross-sectional design, or meta-analysis that clearly described the definition of “early-onset” of AGA. Individuals without alopecia or with late-onset AGA were used as controls. Studies were also included if the definition of “early-onset” was not explicitly described but all the AGA cases involved patients under the age of 40 (the maximum age limit determined according to the initial literature review) and stratified analysis was conducted based on gender, alopecia severity, or other case features; 2) Articles had to be published in English; 3) There were no restrictions on gender or the year of publication.

The following types of papers were excluded from this scoping review: 1) letters, editorials, commentaries, unpublished manuscripts, dissertations, government reports, books and book chapters, conference proceedings, meeting abstracts, lectures and addresses, consensus development statements; 2) case reports; 3) case series; 4) qualitative studies; 5) non-systematic reviews; 6) guideline statements; 7) protocols for proposed systematic reviews or meta-analyses; 8) animal studies; 9) cell culture studies; 10) studies on the efficiency and side effects of AGA treatment.

The literature search was performed by one investigator (Li-Ping Liu). After the duplicates were removed, two reviewers (Li-Ping Liu and Mary Adumo Wariboko) screened all titles, abstracts and full texts independently based on the inclusion and exclusion criteria and solved disagreements by discussion.

### Data extraction

The aim of this review is to systematically map and aggregate the available evidence, not to critically analyzing the quality of individual studies. Therefore, a descriptive analysis was performed to examine the characteristics of the included literature. Data extracted were summarized in a narrative report encompassing the following themes: study characteristics and methodologies (including year of publication, study region, research design, participant characteristics); findings related to the research questions (including main outcomes and study results). The key information and relevant variables were summarized in a descriptive table when necessary. Microsoft Excel and GraphPad Prism were employed for data analysis and graphing purposes.

## Results

### Study selection and data extraction

The search produced 1,147 records (MEDLINE: n = 395, EMBASE: n = 590, CENTRIAL: n = 162) (Fig 1). When duplicated studies (n = 376) were removed, 771 studies were used for further screening. Studies were excluded if there were irrelevant (n = 623), conference abstract (n = 21), non-systematic reviews (n = 15), case reports or case series (n = 11), commentary (n = 1), lacking an appropriate control group (n = 26), non-English (n = 3), or focused on treatment side effects (n = 6). Ultimately, 65 eligible articles were identified.

### Characteristics of included studies

**Fig 2:**
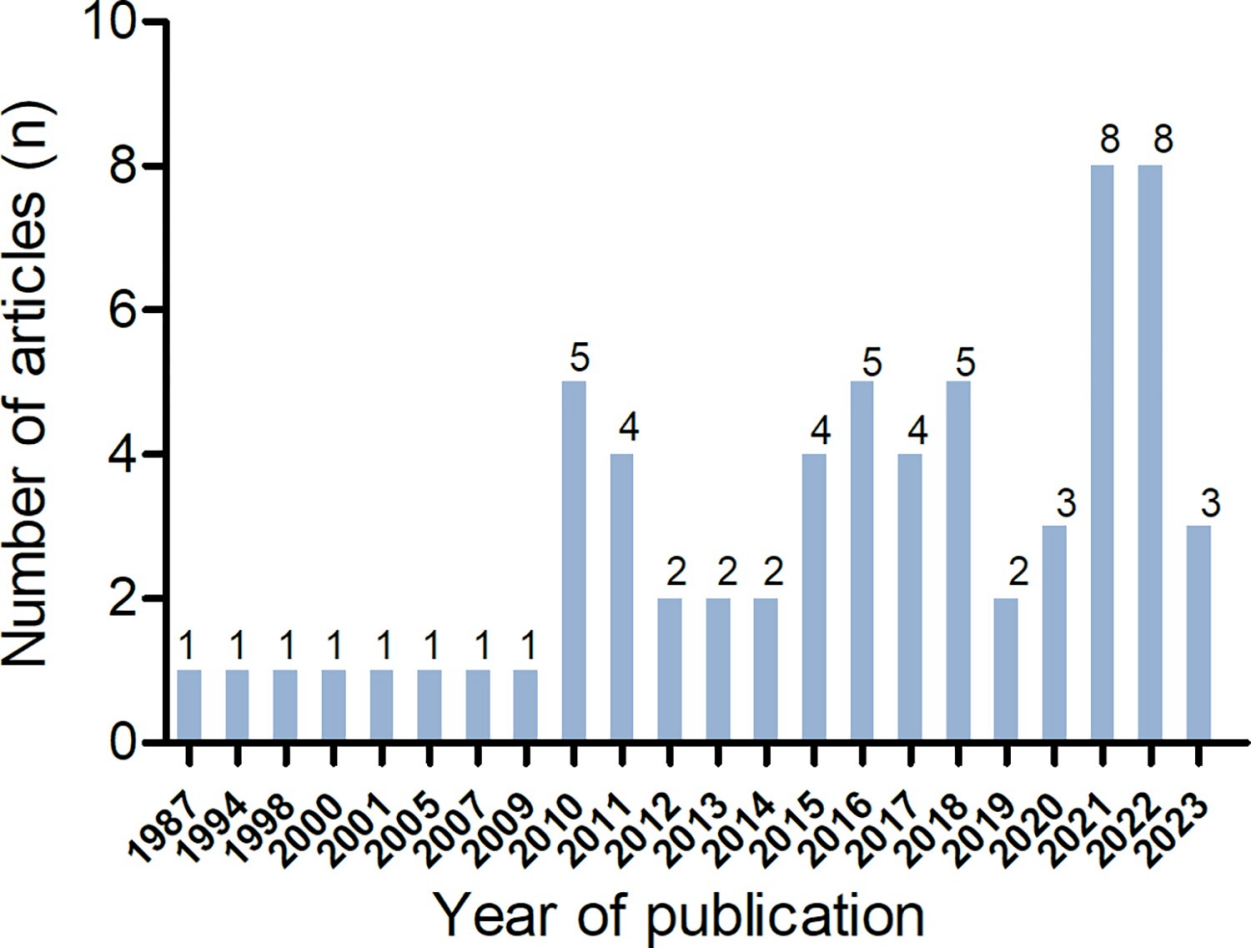
Number of articles published by year.

**Fig 3:**
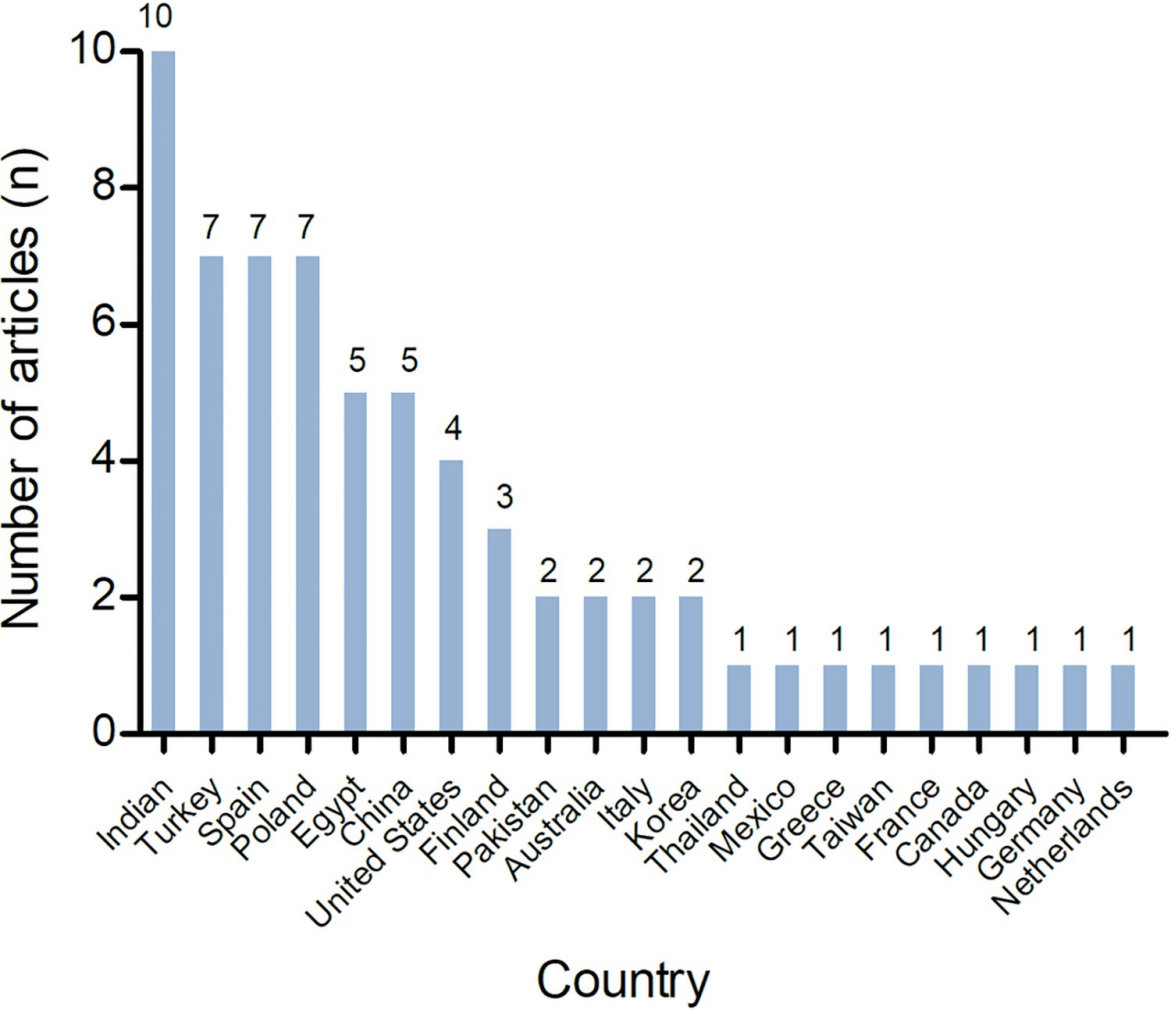
Number of articles published by country.

The majority of the studies included in this scoping review were conducted using a case-control design (67.69%, n = 44). Some studies utilized a cross-sectional design (24.62%, n = 16), cohort design (1.54%, n = 1), or were systematic reviews and meta-analyses (6.15%, n = 4). The number of published articles showed a significant increase after 2020 (Fig 2). The studies originated from 21 different countries, with the highest number of research studies conducted in India (Fig 3). The majority of studies only included male participants (78.46%, n = 51).

### Definition of early-onset AGA and methods for severity evaluation

**Fig 4:**
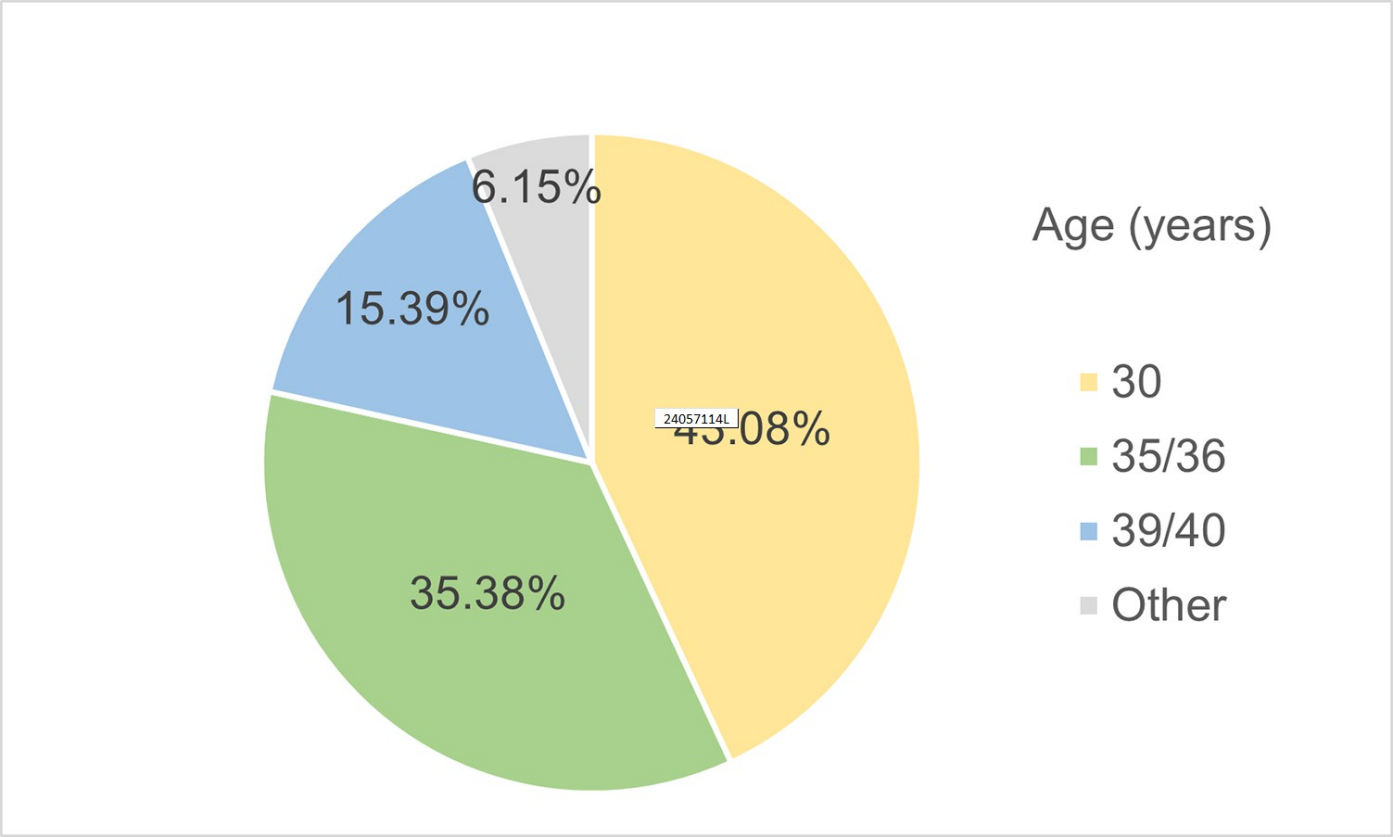
Percentage of studies using age limitation for early-onset AGA.

Among the included studies, 43.08% (n = 28) defined “early-onset AGA” as cases developing before the age of 30 years, while 35.38% (n = 23) included cases developing before 35 or 36 years (Fig 4). The methods applied to evaluate the severity of baldness varied. The HNS was the most frequently used in men (69.23%, n = 45) while the Ludwig scale was predominantly used most in women (10.77%, n = 7). In addition, the basic and specific classification (BASP) scale and the Ebling scale were each used in 4 studies, respectively.

### Topics of included studies

To address the three research questions mentioned above, the topics covered in the included studies were classified into four main categories: investigation of risk factors and etiology (23.08%, n = 15), association with potential comorbidities (61.54%, n = 40), medication effects related to early-onset AGA (10.77%, n = 7) and the impacts of alopecia on patient wellbeing (6.15%, n = 4). The last two categories focused on the potential adverse effects of early-onset AGA on patient wellbeing, with a total of 11 studies addressing this aspect.

### Risk factors and etiology of early-onset AGA

Fifteen articles focused on identifying the risk factors [5, 10, 12, 21–23] or etiology [4, 8, 24–30] for early-onset AGA. Several risk factors were found to be significantly associated with early-onset AGA. These included family history [10, 12, 23], lifestyle factors such as cigarette smoking [10, 21] and unhealthy dietary habits [10]. Additionally, severe AGA was more prevalent in men aged between 30 and 40 years with higher body mass index (BMI) [22]. For young female cases aged 15 to 18 years, predictors of early-onset AGA included irregular menses, a history of thyroid disease, and hirsutism [12].

Two studies investigated the hormonal profile of men with early-onset AGA [4, 24], considering that AGA is an androgen-mediated disease [1]. Sanke S, et al. [4] found that men with early-onset AGA had significantly elevated levels of testosterone, dehydroepiandrosterone sulfate (DHEAS), luteinizing hormone (LH), prolactin, free androgen index and LH/follicle-stimulating hormone (FSH) ratio, while mean levels of FSH and sex hormone binding globulin (SHBG) were decreased. These findings suggest that early-onset AGA in men could be considered as a phenotypic equivalent of polycystic ovary syndrome (PCOS) in women, as it causes similar hormonal changes. However, another study showed similar levels of LH, FSH and SHBG between AGA cases and the control group, although levels of serum free testosterone and dihydrotestosterone were significantly higher in AGA patients [24]. Therefore, the consistency of LH and FSH levels appears to be inconclusive.

Because excessive activity of 5α-reductase in hair follicles is believed to be a contributing factor to AGA [31], the role of this enzyme in early-onset cases was evaluated in three studies. Among hyperandrogenic women, increased 5α-reductase activity in the scalp was found to be associated with alopecia [25]. However, no statistical significance was observed in the mean mRNA levels of three types of 5α-reductase isozymes in plucked hairs between women with AGA and controls [26]. Furthermore, no significant differences were found in allele, genotype, or haplotype frequencies for restriction fragment length polymorphisms of 5 α-reductase enzyme genes (SRD5A1 and SRD5A2) between young bald men and older non-bald men [27].

Genetic predisposition plays a crucial role in the development of AGA. Hillmer AM, et al. [28] explored the contribution of the genetic variability in androgen receptors and found that it was the cardinal prerequisite to the development of early-onset AGA. Additionally, six susceptibility loci (rs12565727, rs9287638, rs2073963, rs6945541, s12373124, rs10502861) and two AGA loci on the X chromosome and chromosome 20 were identified [8]. Moreover, forty-nine single-nucleotide polymorphisms (SNPs) located around PPARGC1A, ABCC4, CYP11B2, FSHB, and CYP19A1 were found to be significant contributors to AGA [29]. Furthermore, a genetic model was established to predict AGA risk based on SNPs identified from previous genome-wide association study [30]. The model achieved an accuracy level of 0.74 in predicting the risk of early-onset AGA. It was found that 55.8% of the genetic liability for early-onset AGA can be attributed to common autosomal SNPs, while 23.3% is attributed to X-chromosome SNPs.

### Conditions associated with early-onset AGA

**Fig 5:**
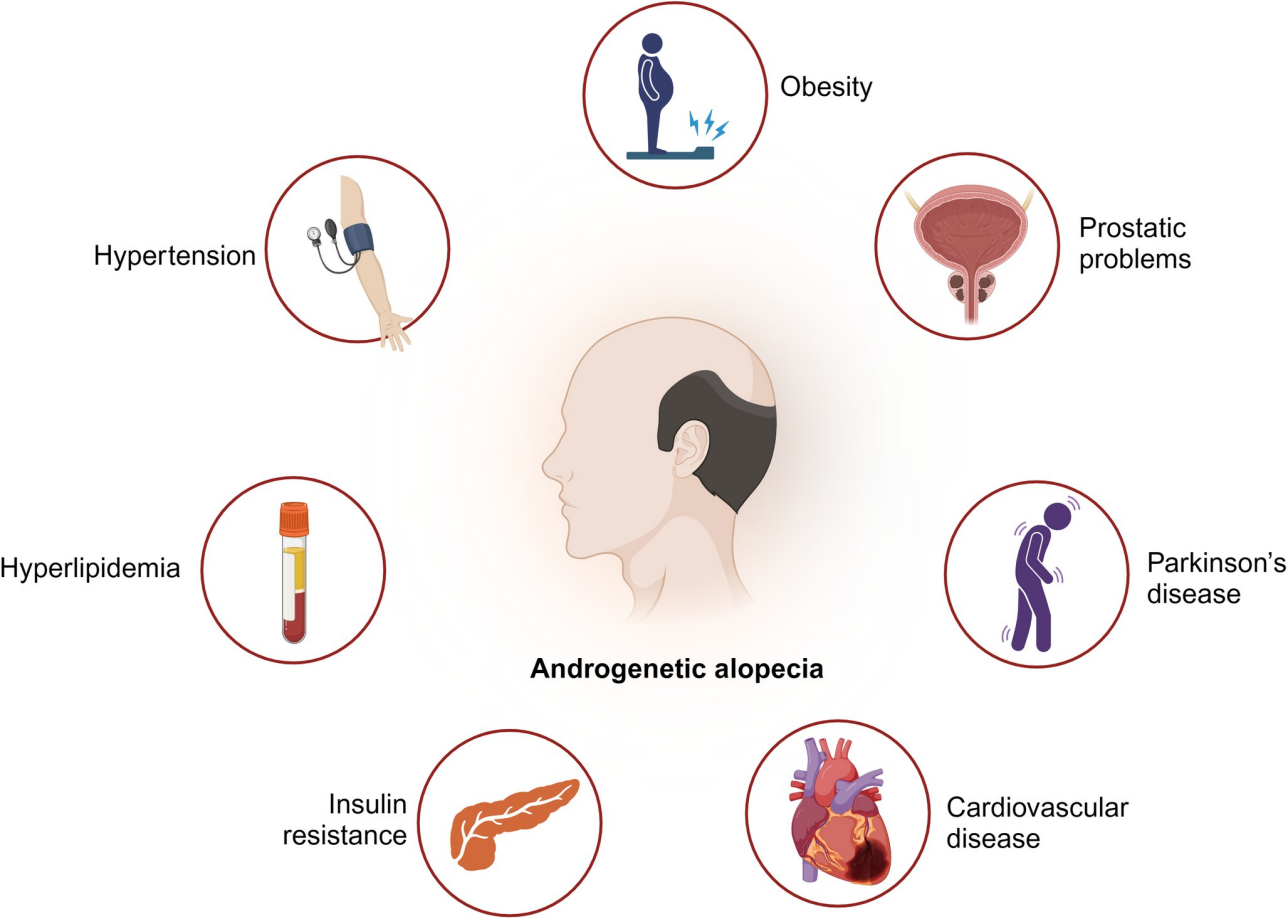
Potential conditions associated with early-onset AGA. (Created with BioRender.com).

A total of 40 articles examined the potential comorbidities associated with early-onset AGA (Fig 5). The majority of them focused on the metabolic diseases (n = 30) [6, 7, 9, 15, 17, 18, 32–55], such as MetS, cardiovascular disease (CVD), IR, dyslipidemia. Other associated conditions included prostatic hyperplasia or prostatic cancer (PC) [19, 56–58] (n = 4), hyperuricemia [59, 60] (n = 2), gonadal and adrenal related problems [61, 62] (n = 2), amyotrophic lateral sclerosis [63] (n = 1), and Parkinson’s disease and fertility [8] (n = 1) (Table 1).

**Table 1:** Conditions associated with early-onset AGA.

Study design	Publication and location	Objective	Age limitation of “early-onset” & severity evaluation method	Participants characteristics	Results	Summary findings
Metabolic diseases						
Systematic Review and meta-analysis	2022 [15]; China	To evaluate the risk grade of MetS and the metabolic profile in patients with early-onset AGA	age < 36 years; N/A	503 with AGA and 467 without AGA; 880 male and 90 female	The pooled ORs for the prevalence rate of MetS between the group without and with early-onset AGA were 3.13 and 3.88, respectively (*P*<0.001).	Patients with early-onset AGA had significant associations with MetS.
	2017 [51]; Italy	To evaluate the difference in the glycolipid and hormonal profiles in men with early-onset AGA compared to controls	age < 35 years; HNS	522 with AGA and 487 without AGA; male	Men with early-onset AGA have significant differences in glycemic profile (higher insulin serum and HOMA index), lipid profile (higher serum TC, LDL cholesterol and triglycerides), and hormonal profile (higher levels of LH and DHEAS, lower levels of SHBG).	Early-onset AGA might represent a phenotypic sign of the male PCOS-equivalent.
Case-control	2022 [35]; India	To compare the prevalence of MetS in early-onset AGA patients	age < 35 years; N/A	50 with AGA and 50 without AGA; 20 to 50 years of age; both sex	1. Two groups did not vary significantly in height and weight, glucose levels, thyroid hormone levels, blood counts, and cholesterol levels. 2. 26% of patients in AGA group and 6% of patients in the control group fit the criteria of MetS (*P* = 0.007). 3. The difference in LDL and HDL values between patients and controls was significant (*P* = 0.0027 and 0.0091 respectively).	Patients with early-onset AGA are more likely to develop MetS.
	2021 [36]; Pakistan	To evaluate the association of early-onset AGA and MetS in young male	age < 36 years; HNS grade ≥ 3	101 with AGA and 101 without AGA; 20–36 years of age; male	There were significant differences in the mean values of serum triglycerides (*P* = 0.006) and WC (*P* = 0.002).	A significant association was found between AGA and MetS.
	2020 [37]; Egypt	To detect the role of serum YKL-40 in early-onset AGA and MetS pathogenesis, onset and severity	age < 30 years; HNS for males and Ludwig for females	70 with AGA and 30 without AGA; 18 to 50 years of age; both sex	1. Patients showed highly significant higher serum YKL-40 level more than those without AGA (*P*< 0.001). 2. There was highly significant increase in YKL-40 level among early-onset cases compared to late-onset ones (*P*< 0.001).	High serum YKL-40 was considered not only a biomarker of early-onset AGA but also a potential sensitive predictor for early-onset MetS development and severity in patients with early-onset AGA.
	2019 [33]; India	To assess the link between MetS and/or IR in men with early-onset AGA	age < 30 years; HNS	50 with AGA and 50 without AGA; 18 to 24 years of age; male	1. 10% AGA group (5/50) and 4% without AGA group (2/50) had IR (*P* = 0.23). 2. 30% AGA group (15/50) and 8% without AGA group (4/50) had MetS (*P* = 0.005).	Male patients with early-onset AGA did not have IR. MetS was linked to early-onset AGA in male patients.
	2018 [38]; India	To find the relationship between AGA and MetS	age < 30 years; HNS grade ≥2	50 AGA and 50 without AGA; 18 to 50 years of age; male	1. MetS was found in 15 (30%) patients and 6 (12%) controls. 29 patients in case and 16 in controls were dyslipidemia. 2. No difference was found in increased blood pressure and hyperglycemia between two groups.	Patients with early-onset AGA tend to have a higher prevalence of MetS, particularly dyslipidemia.
	2017 [34]; Turkey	To investigate the factors affecting AGA, and the relationship between AGA and IMA.	age <35 years; HNS grade ≥3	50 with AGA and 30 without AGA; 18–35 years of age; male	1. There was no significant difference between participants with AGA and control in BMI, DHEAS, testosterone, lipid profile, TC, HDL, LDL, IR, and IMA. 2. There was a positive correlation between AGA grade and IR (r = 0.296, *P* = 0.037).	There was a positive correlation between AGA stage and IR and there was no significant relationship between AGA and IMA.
	2016 [40]; Turkey	To evaluate cardiovascular risk factors in male patients with early-onset AGA	age < 30 years; HNS	51 with AGA and 17 without AGA; 18 to 55 years of age; male	1. MetS was identified in 25 with AGA and 2 without AGA participants (*P*< 0.05). 2. The carotid intima-media thickness values in patients with vertex pattern AGA were found to be substantially greater than in patients without vertex baldness and controls (*P*<0.05). Patients’ pulse-wave velocity values were considerably greater (*P*< 0.001).	Early-onset AGA alone could be an independent risk factor for CVD and MetS.
	2015 [52]; Egypt	To investigate plasma lipid profile and atherogenic index in non-obese females with AGA	age < 35 years; Ludwig	40 with AGA and 40 without AGA; 15 to 40 years of age; female	1. TC (*P*<0.001), LDL cholesterol (*P* = 0.03), and triglycerides (*P* = 0.001) levels were higher in AGA than in controls. The mean level of HDL cholesterol in AGA was lower than in controls (*P* = 0.008). 2. The atherogenic index of plasma in AGA was greater than in controls (*P*<0.001).	It supports the link between early-onset AGA and an unfavorable lipid profile, as well as cardiovascular risk in females.
	2015 [17]; India	To study the presence of MetS and carotid artery atherosclerosis in men with early-onset AGA	age < 35 years; HNS	100 with AGA and 100 without AGA; 18 to 35 years of age; male	1. 22% patients with early-onset AGA met the MetS criteria, compared to 8% in control (*P* < 0.05). 2. There were significant differences in values of WC, serum triglycerides, serum cholesterol, SBP, DBP, fasting glucose concentration, and very LDL between participants with and without AGA. 3. The mean levels of HDL and LDL cholesterol showed no significant changes. 4. The atherosclerotic plaque was discovered in two of the AGA, but not in any of the control.	AGA can be considered as an early indicator of MetS.
	2015 [18]; Egypt	To predict the relation between early AGA and ischemic heart disease	age < 30 years; N/A	100 with AGA and 100 without AGA; 20–30 years of age; male	1. The BMI and WC of AGA group were significantly higher compared with control. 2. Statistically significant higher level in SBP, TG in AGA group, while no significant differences in DBP, HDL, LDL between two groups. 3. ECG found abnormal finding in AGA patient which was statistically significant.	AGA could predict ischemic heart disease.
	2014 [41]; India	To assess the prevalence of MetS in persons with early AGA in Indian settings.	age < 30 years; HNS	85 with AGA and 85 without AGA; less than 30 years of age; male	1. MetS was found in a greater proportion of AGA group (43.5%) than in control (2.4%) (*P*<0.001). 2. AGA group exhibited higher levels of triglyceride (*P*<0.001), SBP (*P*<0.001), DBP (*P*<0.001), and considerably lower HDL levels (*P*<0.001).	MetS is linked to AGA in male Indian patients less than the age of 30.
	2013 [42]; Thailand	To evaluate the relationship between early-onset AGA and MetS in Thai male patients.	age < 35 years; HNS stage ≥ 3	40 with AGA and 40 without AGA; less than 35 years of age; male	1. Early-onset AGA participants showed a 3.48-fold greater risk of MetS than control (*P* = 0.015). 2. AGA severity and MetS had no connection.	A link between early-onset AGA and MetS in Thai males was found.
	2011 [50]; Spain	To evaluate SHBG and blood glucose levels in patients with early-onset AGA	age < 35 years; Ludwig degree ≥II for females and Ebling degree ≥ III for males	120 with AGA and 120 without AGA; 45 to 60 years of age; both sex	1. 39.1% of AGA participants had hyperglycemia compared to 12.5% of those without AGA (*P*<0.0001). 2. SHBG levels in patients with AGA and hyperglycemia were lower than in those without AGA with hyperglycemia (*P* = 0.004). 3. A robust connection between SHBG levels and glucose levels in AGA participants (OR = 3.35, *P*<0.001).	A link between early-onset AGA, hyperglycemia/diabetes, and low SHBG levels was found. In AGA patients, low SHBG levels may be a sign of IR and hyperglycemia / diabetes.
	2011 [47]; Turkey	To identify the presence of IR and MetS in early-onset AGA	age < 30 years; HNS	50 with AGA and 40 without AGA; 18 to 30 years of age; male	1. Values of DBP, TC, HOMA-IR and FIRI were significantly higher in the AGA group. 2. No significant differences were observed in MetS diagnosis between the two groups.	Male patients with early-onset AGA exhibit IR.
	2010 [43]; Turkey	To examine the frequency of IR, hyperinsulinemia and MetS in early-onset AGA.	age < 35 years; HNS	80 AGA and 48 without AGA; 20 to 50 years of age; male	1. The occurrence of MetS was significantly higher in the AGA group than in the control group (*P*< 0.05). 2. IR was detected in 25 patients with early AGA and in 6 healthy participants (*P*< 0.05).	The prevalence of IR and MetS was shown to be higher in early AGA patients.
	2010 [53]; Spain	To analyze the lipid values in men and women with AGA	age <35 years; Ebling degree ≥ III for males and Ludwig degree ≥II for females	150 with AGA and 150 without AGA; 35 to 60 years of age; both	1. Female with AGA showed significant higher values of triglycerides (*P* = 0.006), TC (*P* = 0.014), LDL cholesterol (*P* = 0.0006) and lower HDL cholesterol (*P*<0.0001) versus group without AGA respectively. 2. Men with AGA had significantly higher triglyceride levels (*P* = 0.04), TC levels (*P* = 0.006), and LDL-C levels (*P* = 0.0013).	A significantly higher lipid levels in men and women with AGA than those without AGA was found.
	2010 [7]; Spain	To evaluate aldosterone levels and the presence of systolic and diastolic hypertension in women with early-onset AGA	age < 35 years; Ludwig degree ≥ II	40 with AGA and 40 without AGA; 35–55 years of age; female	Patients with AGA had higher SBP (*P*<0.0001), DBP (*P*<0.0001), and aldosterone levels (*P* = 0.002) than those without AGA.	Women with AGA have a greater frequency of hypertension.
	2010 [44]; Spain	To analyze the presence of cardiovascular risk factors and the prevalence of carotid atheromatosis, hormonal factors, and acute phase reactant variables in AGA patients	age <35 years; Ebling stage ≥ III for males and Ludwig stage ≥ II for females	77 with AGA and 77 without AGA; 35 to 55 years of age; both sex	1. MetS was found in 60% of male AGA (OR = 10.5), 48.6% of female AGA (OR = 10.73), 12.5% of male without AGA, and 8.1% of female without AGA (*P*<0.0001). 2. Atheromatous plaques were seen in 32.5% of male AGA patients (OR = 5.93) compared 7.5% of male without AGA (*P* = 0.005) and 27% of female AGA patients (OR = 4.19) versus 8.1% of female without AGA (*P* = 0.032).	The evaluation of MetS and an ultrasound examination of the carotid arteries may be valuable screening procedures for detecting the risk of developing CVD in patients with early-onset AGA.
	2009 [48]; Mexico	To study the link between AGA and IR using the HOMA-IR index or clinical signs of the MetS	age < 35 years; HNS stage ≥ III	80 with AGA and 80 without AGA; 18–35 years of age; male	1. The HOMA-IR index was significantly higher in cases than controls. 2. Nonobese AGA cases had a higher mean DBP and a more frequent family history of AGA than nonobese controls without AGA.	All young males with stage III or higher AGA should be evaluated for IR and cardiovascular-related characteristics and diseases.
	2001 [45]; Finland	To test if the early-onset AGA is a risk factor for early severe CAD requiring surgery	age < 35 years; HNS grade ≥ III vertex	85 with a coronary revascularization procedure and 85 age-matched control; 43.5–79 years of age; male	The OR for coronary revascularization under the age of 60 years in men with early-onset AGA was 3.18 compared to males without alopecia or late AGA after multivariate adjustment for the traditional CAD risk factors. The OR at any age was 1.84.	Early-onset AGA is a risk factor for an early-onset of severe coronary heart disease.
	2000 [6]; Finland	To test whether early AGA could be a clinical marker of IR	age <35 years; HNS grade ≥ III vertex	125 with AGA and 104 without AGA; 19–50 years of age; male	Men with early-onset AGA had a strikingly increased risk of hyperinsulinemia and IR-associated disorders such as obesity, hypertension, and dyslipidemia.	Early AGA might be an early indication of IR.
Cross-sectional	2023 [49]; India	To ascertain the clinic-phenotypic features, and the prevalence of hormonal and metabolic alterations of males with early-onset AGA	age < 30 years; HNS grade ≥3	100 with AGA (34 with altered hormonal profile and 66 with normal profile); 18–29 years of age; male	1. 34 of the 100 AGA patients had altered hormonal profiles, whereas 44 and 26 had IR and MetS, respectively. 2. Changes in hormone profiles were linked to IR and severe baldness. 3. Patients with altered hormonal profile who were IR had a substantially greater frequency of severe alopecia (*P* = 0.036)	Subjects with changed hormonal profiles were more likely to exhibit IR and severe grades of AGA.
	2022 [32]; Turkey	To evaluate the cardiovascular and metabolic implications of early-onset AGA	age < 30 years; HNS	41 with AGA and 40 without AGA; 24 to 45 years of age; male	1. ABP, hsCRP, and galectin-3 levels were similar in both groups. 2. In AGA patients, there was a positive relationship between HNS grade with age, BMI, triglyceride levels, and fasting blood glucose levels, daytime pulse wave velocity and nighttime reflection magnitude. There was also a substantial positive connection between hsCRP with BMI and WC, and between galectin-3 with BMI, WC, hip circumference, and HOMA-IR.	AGA patients have IR and MetS components similar to the general population. hsCRP and galectin-3 appear to be linked to CVD risk factors in AGA patients.
	2021 [9]; China	To investigate the relationship between AGA and CAD in the Han Chinese male population.	age < 40 years; BASP	402 with suspected or confirmed CAD; 28 to 75 years of age; male	1. Patients with various levels of AGA had varying CAD status (*P* = 0.002), dyslipidemia status (*P* = 0.002), age (*P* = 0.003), and coronary atherosclerosis severity (*P*<0.001). 2. Early-onset AGA was associated with a relative CAD risk of 2.292, while severe AGA was associated with a relative CAD risk of 2.111 (*P* = 0.016). 3. The severity of coronary atherosclerosis was independently correlated with AGA status (OR = 2.247, *P* = 0.001), severe AGA (OR = 2.360, *P*<0.001), and early-onset AGA (OR = 3.474, *P*<0.001).	AGA is independently related with CAD in a Han Chinese male sample.
	2017 [46]; Greece	To evaluate the connection between hypertension and target organ damage in hypertension males with and without AGA	age < 25 years; HNS	101 with consecutive hypertension (62 with AGA and 39 without AGA); 35–52 years of age; male	1. CFRd was considerably lower in severe AGA as compared to mild to moderate AGA participants (*P* = 0.007) and non-AGA participants (*P* = 0.02). 2. AGA severity was independently associated to CFRd and pulse pressure, whereas AGA length and age of initiation were connected to CFRd and pulse pressure, respectively	Hypertension individuals with severe and early AGA development appear to be at increased cardiovascular risk.
	2016 [39]; India	To study the link between MetS and early-onset AGA	age < 35 years; HNS	85 with AGA and 85 without AGA; 18 to 55 years of age; male	1. MetS was observed in 22.4% of the AGA patients and 9.4% of the controls (*P* = 0.021). 2. Patients with AGA had substantially greater levels of abdominal obesity, hypertension, and lowered HDL compared to controls.	Men with early-onset AGA had a greater frequency of MetS.
	2014 [54]; Taiwan	To determine association between BMI and alopecia severity in men with AGA	age < 30 years; HNS	189 with AGA; 18 to 64 years of age; male	1. Men with severe AGA had higher BMI than those with mild to moderate alopecia (*P* = 0.01). 2. The risk for severe alopecia was higher in the overweight or obese subjects with AGA (OR = 3.52, *P*<0.01). 3. In early-onset AGA, the risk for having severe alopecia was higher in the overweight or obese subjects (OR = 4.97, *P* = 0.03).	Higher BMI was significantly associated with greater severity of hair loss in men with AGA, especially with early-onset AGA.
	2007 [55]; Finland	To examine the link between early-onset AGA and low-grade inflammation	age < 35 years; HNS	727; 25–34 years of age; male	1. Men with moderate to extensive AGA (17%) had a higher BMI and larger circumference of upper arm, waist and hip than those with little to no alopecia (*P*<0.05). 2. The mean waist-to-hip circumference ratio increased with rising hsCRP, but only among males with moderate to severe AGA (*P* = 0.043).	A link between moderate to severe AGA and low-grade inflammation was a predictor of future CVD, especially in males with central obesity.
Prostate related						
Case-control	2018 [56]; Australia	To evaluate the associations between AGA at a young age and the development of aggressive PC.	age < 40 years; HNS	1,107 with AGA and 834 without AGA; 20–40 years of age; male	1. Men with very early-onset balding at age 20 years were at increased risk for subsequent aggressive PC (OR = 1.51). 2. The impact was strongest in males with advanced stage pT3+ disease (OR = 1.68), but men with organ-confined high-grade PC did not show the similar pattern.	Men with AGA at the age of 20 are more likely to develop advanced stage PC.
	2012 [19]; Spain	To determine whether individuals with early-onset AGA had larger prostatic volumes and urine flow alterations than healthy subjects without AGA.	age < 35 years; Ebling scale type III, IV, or V	45 with AGA and 42 without AGA; 35–65 years of age; male	1. Patients with AGA had significantly higher mean prostate volume (*P*<0.0001), IPSS (*P*<0.0001), and prostate-specific antigen value (*P*<0.0001), but significantly lower maximum urinary flow (*P*<0.0001). 2. A strong association between the presence of AGA and benign prostatic hyperplasia (OR = 5.14, *P* = 0.041).	The presence of AGA is related with prostate growth-associated urine symptoms. Early-onset AGA may be an early indicator of urinary/prostatic symptomatology.
	2011 [58]; France	To study link between early-onset AGA and risk of PC later in life	age < 40 years; HNS	388 with PC and 281 without PC; The mean age for cases was 67.2±7.2 years, while for controls it was 66.4±9.1 years.; male	1. Patients with PC were twice as likely to have AGA at age 20 (OR = 2.01, *P* = 0.0285). 2. There was no link seen between early-onset AGA and earlier PC detection or the development of more aggressive tumors.	A link between early-onset AGA and PC development was found.
	2010 [57]; United States	To explore the relationship between AGA and PC risk	age < 30 years; Self-defined	999 with PC and 942 without PC; 35–74 years of age; male	1. Hair loss at age 30 was more common in controls (25.2%) than cases (19.8%, *P* = 0.005), and those with hair loss at age 30 had a 29% relative risk reduction for PC (OR = 0.71). 2. In men aged over 60, the risk reduction was greater for men with hair loss at age 30 from both the top of head and forehead (OR = 0.55)	Early-onset AGA was linked with a lower relative risk of PC.
Hyperuricemia						
Case-control	2022 [59]; India	To relate the prevalence of hyperuricemia and level of SUA levels in patients of early onset AGA	age < 35 years; BASP	104 with AGA and 104 without AGA; 18–35 years of age; male	1. SUA was higher in AGA group than in control (*P* = 0.2). 2. AGA patients had more hyperuricemia compared to the group without AGA (*P* = 0.03, OR = 0.39).	Males with early-onset AGA have higher blood uric acid levels. Hyperuricemia is more prevalent in patients with early-onset AGA than in control.
	2020 [60]; China	To determine relationship between hyperuricemia and AGA in males	age < 30 years; HNS grade ≥3	1312 with AGA and 2624 without AGA; 18–50 years of age; male	1. Men with AGA had higher mean uric acid levels (*P*<0.001) and greater prevalence of hyperuricemia (*P*<0.001) compared to men without AGA. 2. AGA severity and hyperuricemia had no statistically significant relationship (*P* = 0.295).	Men with early AGA have a higher prevalence of hyperuricemia.
Gonadal and adrenal related						
Case-control	2020 [61]; Italy	To assess gonadal and adrenal function in males with early-onset AGA	age < 35 years; N/A	43 with AGA and 36 without AGA; 14–30 years of age; male	1. Patients with early-onset AGA exhibited higher BMI (*P*<0.05) and 17α-hydroxyprogesterone (*P*<0.05). 2. Men with early-onset AGA with at least one of the following factors: BMI >25 kg/m^2^, IR, and SHBG<25 nmol/L have higher DHEAS levels and worse gonadal steroidogenesis.	Men with early-onset AGA may have a greater risk to develop gonadal dysfunction later in life.
	1987 [62]; United States	To describe link between early AGA with adrenal hyperactivity	age < 35 years; N/A	18 with AGA and 7 without AGA; 18–35 years of age; male	1. The difference in dehydroepiandrosterone sulphate levels between patients and controls was significant (*P*<0.005). 2. There was no statistically significant difference in serum testosterone values between patients and controls.	Hyperadrenalism may be an important element in the complex biochemistry of AGA.
ALS						
Cohort	2013 [63]; United States	To explore the association of early-onset alopecia and ALS in men	age < 45 years; HNS	42 men with ALS; 46–81 years of age; male	1. Participants with severe alopecia were nearly three times more likely to develop ALS than those with no alopecia (relative risk = 2.74). 2. A strong linear trend between increasing degrees of baldness at 45 years of age and the probability of ALS (*P* = 0.02).	An association between early-onset AGA and ALS was found.
Parkinson’s disease and fertility						
Meta-analysis	2012 [8]; Canada	To detect the association between early-onset AGA and the risk of PD	age < 40 years; N/A	3,891 with AGA and 8,915 without AGA; 46–81 years of age; N/A	1. Variants at 17q21.31 locus including the *MAPT* gene are associated with both risk of PD (*P* = 2.8×10^−12^) and early onset AGA (*P* = 9.3×10^−8^). 2. Early-onset AGA cases had significantly increased odds of subsequent PD (OR = 1.28, *P* = 8.9×10^−3^). 3. The AGA susceptibility alleles at the 17q21.31 locus on the H1 haplotype has been linked to decreased fertility. Individuals in the highest risk quartile of a genotype score had an approximately six-fold increased risk of early-onset AGA (OR = 5.78, *P* = 1.4×10^−88^).	Associations between early-onset AGA, Parkinson’s disease, and decreased fertility was found.

Abbreviation: AGA: androgenetic alopecia; MetS: metabolic syndrome; N/A: not applicable; OR: odds ratio; HNS: Hamilton-Norwood scale; HOMA: homeostatic model assessment; TC: total cholesterol; LDL: low density lipoprotein; LH: luteinizing hormone; DHEAS: dehydroepiandrosterone sulfate; SHBG: sex hormone binding globulin; PCOS: polycystic ovary syndrome; ABP: ambulatory blood pressure; hsCRP: high-sensitivity C-reactive protein; BMI: body mass index; WC: waist circumference; IR: insulin resistance; CVD: cardiovascular disease; HDL: high density lipoprotein; YKL: tyrosine (Y), lysine (K) and leucine (L); IMA: ischemia modified albumin; SBP: systolic blood pressures; DBP: diastolic blood pressures; TG: triglycerides; ECG: electrocardiography; FIRI: fasting insulin resistance index; CAD: coronary artery disease; BASP: basic and specific classification; CFRd: coronary flow reserve; PC: prostate cancer; IPSS: International Prostate Symptom Score; SUA: serum uric acid; ALS: amyotrophic lateral sclerosis; PD: Parkinson’s disease

While some studies did not find significant difference between AGA patients and healthy controls in terms of MetS risk [32], IR [32–34], lipid profile [34], and BMI [34], the majority of studies (72.50%, n = 29) reported that patients with early-onset AGA had a higher risk to develop MetS [15, 17, 33, 35–44], CVD [9, 18, 40, 44–46], IR [6, 43, 47–49], hyperglycemia [50], dyslipidemia [38, 51–53], hypertension [7], high BMI and obesity [18, 54, 55, 61]. A systematic review including nine studies and 970 participants found that a younger onset age (before 36 years) was associated with a higher risk with MetS, with a pooled OR of 3.88 (*P*<0.001) [15]. Moreover, positive associations were also found between AGA severity and the risk of metabolic diseases [9, 32, 34, 46, 48, 49, 54, 55] in all but one study [42]. Two other systematic reviews indicated that early-onset cases might represent a phenotypic sign of male PCOS-equivalent [51] and be associated with an increased risk of developing Parkinson’s disease [8]. Four studies focused on the link between early-onset AGA and PC development, but their results varied greatly [19, 56–58].

### Drug effects

Seven articles, conducted by the same research group in Poland, investigated whether the presence of early-onset AGA altered the efficacy of specific medications for specific disorders (Table 2). These were all case-control studies. The results showed that men with early-onset AGA may experience less benefit from rosuvastatin [64], metformin [65, 66], lisinopril [67], levothyroxine [68], Vitamin D [69], or bromocriptine [70] when treating dyslipidemia, prediabetes, hypertension, autoimmune hypothyroidism, autoimmune thyroiditis, or prolactin excess, respectively, compared to the contemporaries without alopecia. However, one exception was noted, as the impact of metformin on gonadotroph secretory function appeared to be stronger in men with early-onset AGA compared to those with normal hair growth [66].

**Table 2:** Early-onset AGA modifies drug effects.

Target disease & medication	Publication and location	Objective	Age limitation of “early-onset” & severity evaluation method	Participants characteristics	Results	Summary findings
Dyslipidemia treated by Rosuvastatin	2021 [64]; Poland	To investigate levels of cardiometabolic risk factors in HMG-CoA-treated men with early-onset AGA.	age < 30 years; HNS grade ≥ III vertex;	Patients with mixed dyslipidemia: 25 with AGA and 25 without AGA; 18 to 35 years of age; male	1. Rosuvastatin reduced TC, LDL cholesterol, hsCRP, and fibrinogen in both groups and the effects were more pronounced in group without AGA. 2. Rosuvastatin deteriorate insulin sensitivity only in group with AGA and the drug affect uric acid, homocysteine, and 25-hydroxyvitamin D only in group without AGA.	Males with early-onset AGA may benefit less from rosuvastatin medication than their contemporaries.
Diabetes treated by metformin	2022 [65]; Poland	To investigate whether the presence of early-onset AGA modulates the metabolic effects of metformin.	age < 30 years; HNS grade ≥ III vertex	Men at high risk for type 2 diabetes: 72 with AGA and 75 without AGA; 25 to 50 years of age; male	1. Twelve-month metformin treatment reduced fat content, WC, glycated hemoglobin, glucose and triglycerides, as well as improved insulin sensitivity in both group and these effects were more pronounced in group without AGA. 2. Treatment-induced changes in glucose homeostasis markers correlated with the impact of metformin on hsCRP and 25-hydroxyvitamin D levels.	Metabolic effects of metformin are attenuated in men with early-onset AGA.
	2022 [66]; Poland	To determine whether the existence of elevated androgen levels in males with early-onset AGA modulates the impact of metformin on pituitary hormone production	age < 30 years; HNS grade ≥ III vertex	Patients with prediabetes: 23 with early-onset AGA and 25 without AGA; 20 to 50 years of age; male	1. The effect of metformin on fasting glucose, insulin sensitivity, and glycated hemoglobin was more pronounced in group without AGA than in group with AGA. 2. Metformin reduced LH levels and the LH/FSH ratio only in group with AGA.	The impact of metformin on gonadotroph secretory function is stronger in men with early-onset AGA.
Hypertension treated by lisinopril	2021 [67]; Poland	To determine whether early-onset AGA in men with arterial hypertension affects the cardiometabolic consequences of lisinopril.	age < 30 years; HNS grade ≥ III vertex	Patients with grade 1 hypertension: 31 with early-onset AGA and 31 without AGA; 18 to 50 years of age; male	1. Lisinopril reduced systolic and diastolic blood pressure, UACR, hsCRP, and fibrinogen in both groups and the effects were stronger in group without AGA. 2. The drug decreased levels of uric acid and homocysteine, as well as improved insulin sensitivity only in group without AGA. 3. The impact of lisinopril on uric acid, hsCRP, fibrinogen, homocysteine, and UACR correlated weakly with its hypotensive properties, androgen levels, and insulin sensitivity.	Cardiometabolic effects of lisinopril are less pronounced in men with early-onset AGA.
Autoimmune hypothyroidism treated by levothyroxine	2021 [68]; Poland	To examine whether early-onset AGA determines the impact of exogenous levothyroxine on thyroid autoimmunity and hypothalamic–pituitary–thyroid axis activity in young men with autoimmune hypothyroidism.	age < 30 years; HNS grade ≥ III vertex	Patients with autoimmune hypothyroidism: 24 with AGA and 24 without AGA; 18 to 40 years of age; male	Levothyroxine decreased thyroid antibody titers, reduced thyrotropin levels and increased free thyroid hormone levels in both groups and these effects were less pronounced in the men with AGA.	The benefits of levothyroxine therapy in men with autoimmune hypothyroidism are less pronounced in individuals with early-onset AGA
Autoimmune thyroiditis treated by Vitamin D	2021 [69]; Poland	To examine whether early-onset AGA determines the impact of exogenous vitamin D on thyroid autoimmunity and thyroid function in men with autoimmune thyroiditis.	age < 30 years; HNS grade ≥ III vertex	Patients with autoimmune thyroiditis: 25 with early-onset AGA and 23 without AGA; 18 to 35 years of age; male	1. Vitamin D reduced antibody titers in both groups but this effect was stronger in group without AGA. 2. Vitamin D increased SPINA-GT only in group without AGA.	Euthyroid men with early-onset androgenic alopecia may benefit to a lesser degree from vitamin D treatment than other subjects with autoimmune thyroiditis.
Prolactin excess treated by bromocriptine	2023 [70]; Poland	To determine whether early-onset AGA changes bromocriptine’s cardiometabolic consequences in males with prolactin excess.	age < 30 years; HNS grade ≥ III vertex	Patients with prolactin excess: 17 with AGA and 17 without AGA; 20 to 70 years of age; male	1. Bromocriptine reduced prolactin, increased total and bioavailable testosterone, improved insulin sensitivity, and decreased uric acid, hsCRP, and homocysteine in both groups and the impact was stronger in group without AGA. 2. Bromocriptine increase HDL cholesterol and decrease triglycerides, fibrinogen, and UACR only in group without AGA. 3. The impact on cardiometabolic risk factors was inversely correlated with testosterone levels in group with AGA.	Men with early-onset AGA are partially resistant to the cardiometabolic effects of bromocriptine

Abbreviation: AGA: androgenetic alopecia; HMG-CoA: 3-hydroxy-3-methylglutaryl coenzyme A; HNS: Hamilton-Norwood scale; TC: total cholesterol; LDL: low density lipoprotein; hsCRP: high-sensitivity C-reactive protein; WC: waist circumference; LH: luteinizing hormone; FSH: follicle-stimulating hormone; UACR: urinary albumin-to-creatinine ratio; SPINA: structure parameter inference approach; HDL: high density lipoprotein.

### Impacts of early-onset AGA on patient wellbeing

Hair plays a significant role in an individual’s self-image and can affect how one is perceived in social perceptions. A systematic review and meta-analysis of 41 studies with 7,995 patients revealed that AGA was associated with moderate impairment of both health-related quality of life and emotional wellbeing [71]. The detrimental impacts on self-esteem and specific aspects of psychological adjustment are more pronounced in women compared to men [72]. Four included studies evaluated the negative effects of alopecia on young patients [73–76]. However, all of these studies exclusively enrolled male participants. Three of these studies were descriptive cross-sectional studies focused on psychological complications. They found that young men under 30 years old affected by AGA tended to suffer from anxiety and low self-esteem compared to their peers without hair loss [73]. It is also reported that individuals with moderate to severe psychosocial morbidity may have an increased risk of sexual dysfunction [75]. Furthermore, statistically significant associations were observed between the severity of AGA and the severity of depression, loneliness, and levels of internet addiction levels [74].

## Discussion

This scoping review identified several reported risk factors for early-onset AGA, including family history, cigarette smoking, unhealthy dietary habits, high BMI, and irregular menses. Additionally, early-onset AGA may also be associated with hormonal profiles, 5α-reductase enzyme activity, androgen receptor gene, and some newly discovered susceptibility loci. There results highlight the multifactorial nature of the AGA, indicating that a combination of genetic, hormonal, behavioral and lifestyle factors contribute to its development. Additionally, the available studies also demonstrated a close link between early-onset AGA and MetS, CVD, IR, and dyslipidemia. Interestingly, these chronic diseases are also strongly related to the risk factors for early-onset AGA, such as smoking [77, 78] and dietary habits [79–81]. Therefore, this emphasizes the importance of lifestyle modifications in individuals with early-onset AGA to prevent these co-existing health conditions. Furthermore, men with early-onset AGA may benefit to a lesser degree from treatments such as rosuvastatin [64], metformin [65, 66], or lisinopril [67] compared to individuals with normal hair growth. Therefore, young patients with AGA may not only be more susceptible to developing metabolic diseases, but also less responsive to certain treatments. These findings are of clinical significance because AGA can be easily recognized based on clinical features. Tailored treatment approaches may be necessary for this specific population. Overall, the findings underscore the importance of early detection, risk assessment, and comprehensive management of early-onset AGA. Both physicians and patients should consider not only the hair loss condition itself but also its potential associations with other health conditions. Compared to published systematic review and meta-analysis on early-onset AGA, this scoping review provides a more comprehensive understanding of AGA by examining multiple perspectives. It goes beyond focusing solely on a specific factor [51] or the correlation with a specific disease [15], offering a more holistic view of AGA.

Several limitations should be noted. Firstly, our inclusion criteria only considered articles published in English, which may introduce publication bias and limit the generalizability of our findings. Secondly, the quality of included studies were not appraised, which could potentially include studies with lower methodological quality. Thirdly, lack of data synthesis may limit the ability to provide precise statistical information and draw conclusive inferences from the results. Lastly, the heterogeneity in study design, the definition of “early-onset” and methodologies used for AGA evaluation among the included studies can also be considered limitations. The lack of standardized criteria and inconsistency may introduce variability in the findings and make comparisons between studies challenging.

## Conclusion

These findings indicate that early-onset AGA has a complex and multifactorial cause, and it may serve as an indicator of underlying systemic health issues. Furthermore, certain drug treatments may be less effective in men with early-onset AGA, highlighting the importance of considering AGA in the management of these conditions. Additionally, the impact of AGA on psychological well-being should be emphasized in young men.

## Supporting information

S1 Table PRISMA-ScR-Checklist. (DOCX)

S2 Table Search strategy. (DOCX)

S3 Table Dataset of eligible articles. (XLSX)
