# Supplementary material for: Factors associated with early-onset androgenetic alopecia: A scoping review
Source: PLoS One. 2024 Mar 7;19(3):e0299212. doi: 10.1371/journal.pone.0299212 (PMC10919688; doi:10.1371/journal.pone.0299212)
Supplement: S2 Table — (DOCX) [file pone.0299212.s002.docx]

| S2 Table: Database search terms conducted on July 28, 2023  MEDLINE  ("androgenic alopecia"[Title/Abstract] OR "androgenetic alopecia"[Title/Abstract] OR "male pattern hair loss"[Title/Abstract] OR "male pattern baldness"[Title/Abstract] OR "female pattern hair loss"[Title/Abstract] OR "female pattern baldness"[Title/Abstract]) AND ("early-onset"[Title/Abstract] OR "early"[Title/Abstract] OR "young"[Title/Abstract] OR "adolescen*"[Title/Abstract] OR "pediatric*"[Title/Abstract] OR "child*"[Title/Abstract])  EMBASE  #3 #1 AND #2  #2 'early onset':ab,ti OR early:ab,ti OR young:ab,ti OR adolescen*:ab,ti OR pediatric*:ab,ti OR child*:ab,ti  #1 'androgenic alopecia':ab,ti OR 'androgenetic alopecia':ab,ti OR 'male pattern hair loss':ab,ti OR 'male pattern baldness':ab,ti OR 'female pattern hair loss':ab,ti OR 'female pattern baldness':ab,ti  CENTRAL  #3 #1 AND #2  #1 “androgenic alopecia” OR “androgenetic alopecia” OR “male pattern hair loss” OR “male pattern baldness” OR “female pattern hair loss” OR “female pattern baldness”  #2 early-onset OR early OR young OR adolescen* OR pediatric* OR child* |
| --- |
